# New records of six moth (Lepidoptera: Erebidae, Lasiocampidae) species in south African countries, with comments on their distribution

**DOI:** 10.3897/BDJ.8.e59339

**Published:** 2020-11-25

**Authors:** Sylvain Delabye, Ondřej Sedláček, Vincent Maicher, Robert Tropek

**Affiliations:** 1 Institute of Entomology, Biology Centre, Czech Academy of Sciences, Branišovská 31, 37005, České Budějovice, Czech Republic Institute of Entomology, Biology Centre, Czech Academy of Sciences, Branišovská 31, 37005 České Budějovice Czech Republic; 2 Faculty of Science, University of South Bohemia, Branišovská 1760, 37005, České Budějovice, Czech Republic Faculty of Science, University of South Bohemia, Branišovská 1760, 37005 České Budějovice Czech Republic; 3 Department of Ecology, Faculty of Science, Charles University, Vinična 7, 12844, Prague, Czech Republic Department of Ecology, Faculty of Science, Charles University, Vinična 7, 12844 Prague Czech Republic; 4 Nicholas School of the Environment, Duke University, 9 Circuit Dr., NC 27710, Durham, United States of America Nicholas School of the Environment, Duke University, 9 Circuit Dr., NC 27710 Durham United States of America

**Keywords:** Afrotropics, faunistic report, light trapping, savannahs, southern African region, Zambezian region

## Abstract

**Background:**

Southern Africa hosts a high diversity of ecosystems and habitats with a tremendous diversity of Lepidoptera. Although it is one of the most studied parts of the Afrotropics, the knowledge on diversity and distribution of south African moth fauna remains insufficient. To partly fill this gap, we surveyed macromoths by automatic light traps in five localities in two relatively less sampled south African countries.

**New information:**

We reported six species and one genus (*Remigioides*) of moths which had not yet been recorded in Namibia or Zimbabwe. Although none of these records broadened the known distribution of individual species to a new biogeographical region, they still fill important gaps in their distributions. The known distributional ranges of two species have been substantially extended, although they are still within the same biogeographical regions: ca. 800 km southwards for *Remigioides remigina* (Mabille, 1884) and ca. 600 km westwards for *Haplopacha cinerea* Aurivillius, 1905.

## Introduction

The south African countries offer a wide variety of biomes, from deserts and grasslands to woodland savannahs. Some of them are considered to be biodiversity hotspots, such as the Succulent Karoo, the Cape Floristic Province and the Maputaland-Pondoland-Albany (Myers et al. 2000, Mittermeier et al. 2004). Besides these unique ecosystems, most of southern Africa is covered by open and woodland savannahs belonging to two distinct bioregions: the Southern African region and the Zambezian region (Linder et al. 2012). For some taxa (including many groups of plants, mammals and birds), parts of these savannahs are known to harbour a species richness similar to Afrotropical rainforests (Murphy et al. 2016).

However, knowledge on the diversity and distribution of macromoths (Lepidoptera: Macroheterocera, hereinafter referred as *moths*) in these regions is uneven. Due to the long tradition of entomological research, the moth fauna of South Africa is relatively well known, with ca. 7,300 taxa identified in De Prins and De Prins (2020). In comparison, the neighbouring countries of Namibia and Zimbabwe are only represented by ca. 1,500 and 3,000 moth taxa, respectively, in the same database.

Our recent sampling of moths in savannahs of Namibia and Zimbabwe resulted in more than 12,000 trapped individuals. Amongst these, we identified six moth species recorded for the first time in one or the other of the two countries. Here, we present the sampled material of these six species, with remarks on their general distribution and their reported expansion.

## Materials and methods

**Figure 1.**
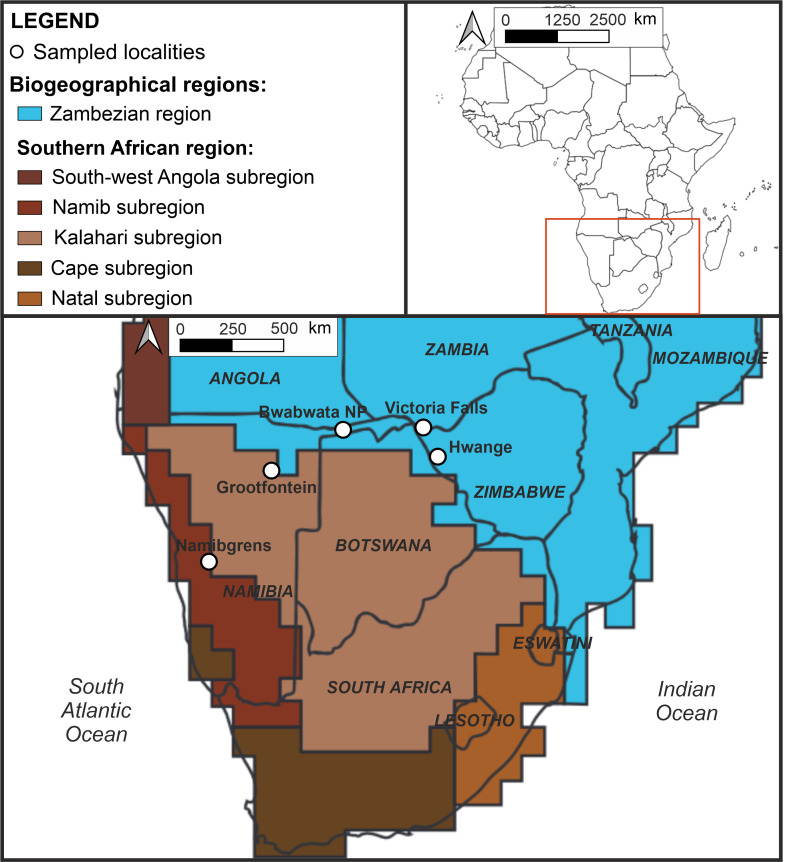
Map of the biogeographical regions and subregions in outhern Africa (modified after Linder et al. 2012), with positions of the study localities.

All reported moth specimens were collected in five localities in Namibia and Zimbabwe (Figs 1, 2, Table 1) in 2016 and 2017. The nomenclature for biogeographical region follows Linder et al. (2012). Vegetation units are based on Burgess et al. (2004), Sayre et al. 2013, Heppner (1991) and Hacker (2019) (Table 1).

All moths were attracted by light. The traps consisted of a two-sided strip of 48 LEDs emitting UV light and powered by 12V batteries placed at the intersection of three plexiglass panels placed on top of a plastic bucket. Attracted specimens were anaesthetised by ammonium hydrogen carbonate. More than 12,000 individuals were trapped. Nine families were focused on (i.e. Erebidae, Eutellidae, Noctuidae, Notodontidae, Eupterotidae, Lasiocampidae, Saturniidae, Sphingidae and Limacodidae) and, after removing non-focused groups (mainly Geometridae), our dataset comprised 9,048 specimens belonging to 488 species or morphospecies, based mostly on external morphology using various available literature, including numerous online resources. Genitalia dissection was done when needed and the exhaustive collection in the Nature Education Centre (Krakow, Poland) was consulted for confirmation of some species identification. From this material, six reliably identified species appeared to be new country records; these are included in this report.

Nomenclature and distribution of the reported species were based on the AfroMoths online database (De Prins and De Prins 2020), on the Global Biodiversity Information Facility data infrastructure (GBIF.org 2020), on the LepiMAP database (LepiMAP 2014) and on the Barcoding of Life Data System (Ratnasingham and Hebert 2007). The voucher material is deposited in the Nature Education Centre of Jagiellonian University in Krakow, Poland.

## Taxon treatments

### Plecoptera sarcistis

Hampson, 1910

8705F178-C4D5-518C-96C3-0F1347230106

Erebidae , Anobinae

#### Materials

- **Type status:** Other material. **Occurrence:** individualCount: 1; lifeStage: adult; **Taxon:** scientificName: Plecoptera sarcistis Hampson, 1910; **Location:** continent: Africa; country: Namibia; stateProvince: Kavango East; locality: Bwabwata National Park; verbatimElevation: 1,018 m; decimalLatitude: -18.1170; decimalLongitude: 21.6797; **Identification:** identifiedBy: Sylvain Delabye; dateIdentified: 2019; **Event:** samplingProtocol: Light-trapping; eventDate: 18/11/2016; **Record Level:** type: PhysicalObject; institutionID: GRBIO URIhttp://grbio.org/cool/8t1f-g2z6; institutionCode: ZMJU; basisOfRecord: PreservedSpecimen
- **Type status:** Other material. **Occurrence:** individualCount: 2; lifeStage: adult; **Taxon:** scientificName: Plecoptera sarcistis Hampson, 1910; **Location:** continent: Africa; country: Namibia; stateProvince: Kavango East; locality: Bwabwata National Park; verbatimElevation: 1,042 m; decimalLatitude: -18.0688; decimalLongitude: 21.6702; **Identification:** identifiedBy: Sylvain Delabye; dateIdentified: 2019; **Event:** samplingProtocol: Light-trapping; eventDate: 20/11/2016; **Record Level:** type: PhysicalObject; institutionID: GRBIO URIhttp://grbio.org/cool/8t1f-g2z6; institutionCode: ZMJU; basisOfRecord: PreservedSpecimen
- **Type status:** Other material. **Occurrence:** individualCount: 1; lifeStage: adult; **Taxon:** scientificName: Plecoptera sarcistis Hampson, 1910; **Location:** continent: Africa; country: Namibia; stateProvince: Kavango East; locality: Bwabwata National Park; verbatimElevation: 1,044 m; decimalLatitude: -18.0592; decimalLongitude: 21.6872; **Identification:** identifiedBy: Sylvain Delabye; dateIdentified: 2019; **Event:** samplingProtocol: Light-trapping; eventDate: 20/11/2016; **Record Level:** type: PhysicalObject; institutionID: GRBIO URIhttp://grbio.org/cool/8t1f-g2z6; institutionCode: ZMJU; basisOfRecord: PreservedSpecimen
- **Type status:** Other material. **Occurrence:** individualCount: 2; lifeStage: adult; **Taxon:** scientificName: Plecoptera sarcistis Hampson, 1910; **Location:** continent: Africa; country: Namibia; stateProvince: Kavango East; locality: Bwabwata National Park; verbatimElevation: 1,051 m; decimalLatitude: -18.0545; decimalLongitude: 21.6954; **Identification:** identifiedBy: Sylvain Delabye; dateIdentified: 2019; **Event:** samplingProtocol: Light-trapping; eventDate: 20/11/2016; **Record Level:** type: PhysicalObject; institutionID: GRBIO URIhttp://grbio.org/cool/8t1f-g2z6; institutionCode: ZMJU; basisOfRecord: PreservedSpecimen
- **Type status:** Other material. **Occurrence:** individualCount: 7; lifeStage: adult; **Taxon:** scientificName: Plecoptera sarcistis Hampson, 1910; **Location:** continent: Africa; country: Namibia; stateProvince: Kavango East; locality: Bwabwata National Park; verbatimElevation: 1,047 m; decimalLatitude: -18.0498; decimalLongitude: 21.7035; **Identification:** identifiedBy: Sylvain Delabye; dateIdentified: 2019; **Event:** samplingProtocol: Light-trapping; eventDate: 20/11/2016; **Record Level:** type: PhysicalObject; institutionID: GRBIO URIhttp://grbio.org/cool/8t1f-g2z6; institutionCode: ZMJU; basisOfRecord: PreservedSpecimen

#### Description

The identification was based on Hampson (1910).

#### Distribution

**Figure 3.**
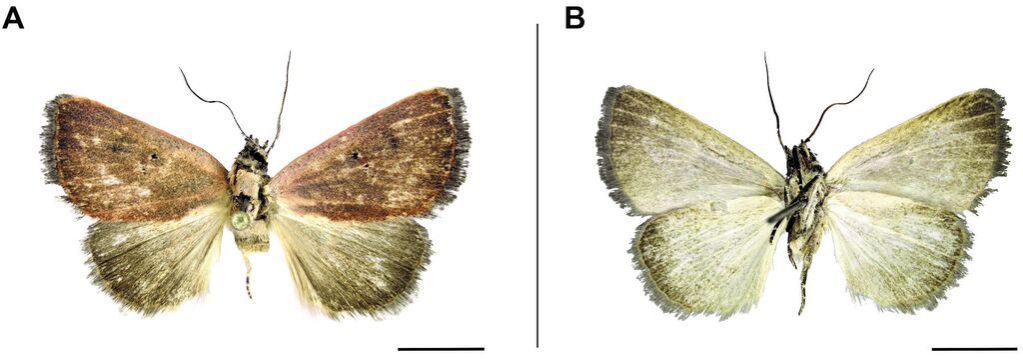
*Plecoptera sarcistis* Hampson, 1910, male, Namibia, Bwabwata National Park. **A.** dorsal view; **B.** ventral view. The scale represents 1 cm.

This species is recorded for the first time in Namibia. All reported specimens were collected in the Bwabwata National Park belonging to the Zambezian biogeographical region. In this region, the species was already known from Zimbabwe and Zambia and it was also already recorded in the neighbouring Southern African region. Therefore, its distribution in north-eastern Namibia could be expected (Fig. 3).

### Hypopyra africana

(Kirby, 1896)

6816229D-D794-545B-A6C5-E31B4973D70C

Erebidae , Erebinae

#### Materials

- **Type status:** Other material. **Occurrence:** individualCount: 2; sex: males; lifeStage: adult; **Taxon:** scientificName: Hypopyra africana (Kirby, 1896); **Location:** continent: Africa; country: Zimbabwe; stateProvince: Matabeleland North; locality: Victoria Falls; verbatimElevation: 927 m; decimalLatitude: -17.9018; decimalLongitude: 25.7634; **Identification:** identifiedBy: Sylvain Delabye; dateIdentified: 2019; **Event:** samplingProtocol: Light-trapping; eventDate: 14/12/2017; **Record Level:** type: PhysicalObject; institutionID: GRBIO URIhttp://grbio.org/cool/8t1f-g2z6; institutionCode: ZMJU; basisOfRecord: PreservedSpecimen
- **Type status:** Other material. **Occurrence:** individualCount: 1; sex: female; lifeStage: adult; **Taxon:** scientificName: Hypopyra africana (Kirby, 1896); **Location:** continent: Africa; country: Zimbabwe; stateProvince: Matabeleland North; locality: Victoria Falls; verbatimElevation: 969 m; decimalLatitude: -17.9099; decimalLongitude: 25.7487; **Identification:** identifiedBy: Sylvain Delabye; dateIdentified: 2019; **Event:** samplingProtocol: Light-trapping; eventDate: 14/12/2017; **Record Level:** type: PhysicalObject; institutionID: GRBIO URIhttp://grbio.org/cool/8t1f-g2z6; institutionCode: ZMJU; basisOfRecord: PreservedSpecimen
- **Type status:** Other material. **Occurrence:** individualCount: 2; sex: males; lifeStage: adult; **Taxon:** scientificName: Hypopyra africana (Kirby, 1896); **Location:** continent: Africa; country: Zimbabwe; stateProvince: Matabeleland North; locality: Victoria Falls; verbatimElevation: 908 m; decimalLatitude: -17.8281; decimalLongitude: 25.6568; **Identification:** identifiedBy: Sylvain Delabye; dateIdentified: 2019; **Event:** samplingProtocol: Light-trapping; eventDate: 13/12/2017; **Record Level:** type: PhysicalObject; institutionID: GRBIO URIhttp://grbio.org/cool/8t1f-g2z6; institutionCode: ZMJU; basisOfRecord: PreservedSpecimen
- **Type status:** Other material. **Occurrence:** individualCount: 1; sex: males; lifeStage: adult; **Taxon:** scientificName: Hypopyra africana (Kirby, 1896); **Location:** continent: Africa; country: Zimbabwe; stateProvince: Matabeleland North; locality: Victoria Falls; verbatimElevation: 909 m; decimalLatitude: -17.8416; decimalLongitude: 25.6446; **Identification:** identifiedBy: Sylvain Delabye; dateIdentified: 2019; **Event:** samplingProtocol: Light-trapping; eventDate: 13/12/2017; **Record Level:** type: PhysicalObject; institutionID: GRBIO URIhttp://grbio.org/cool/8t1f-g2z6; institutionCode: ZMJU; basisOfRecord: PreservedSpecimen

#### Description

The identification of this species was based on photograph of the type specimen in De Prins and De Prins (2020) and comparison with the original description in Kirby (1896).

#### Distribution

**Figure 4.**
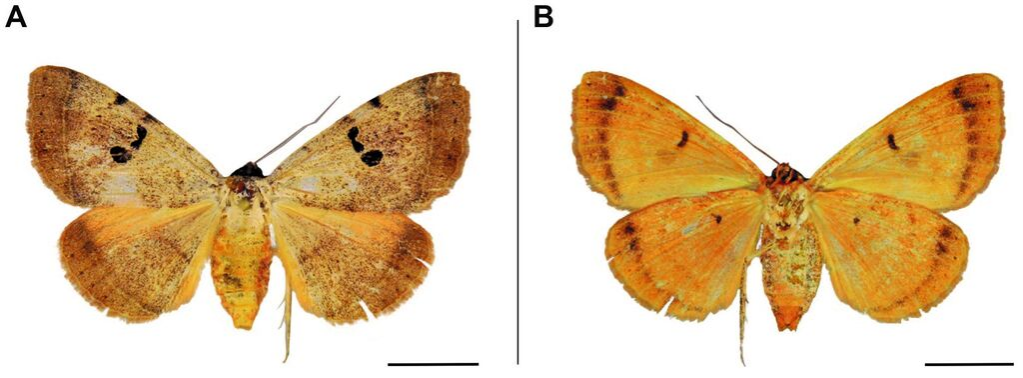
*Hypopyra africana* (Kirby, 1896), female, Zimbabwe, Victoria Falls. **A.** dorsal view; **B.** ventral view. The scale represents 1 cm.

Our records expand the known distribituion of the species to Zimbabwe. *Hypopyra africana* was previously reported from the Somalian (Kenya), Zambezian and Southern African regions, including countries bordering with Zimbabwe (Botswana, Namibia, South Africa, Zambia). All six reported specimens were found near Victoria Falls and partially fill the gap in our knowledge of the distribution of this species (Fig. 4).

### Remigiodes remigina

(Mabille, 1884)

EB9ED5A3-30F0-5214-A3C3-BEA95FB1F126

Erebidae , Erebinae

#### Materials

- **Type status:** Other material. **Occurrence:** individualCount: 3; sex: males; lifeStage: adult; **Taxon:** scientificName: Remigiodes remigina (Mabille, 1884); **Location:** continent: Africa; country: Zimbabwe; stateProvince: Matabeleland North; locality: Hwange; verbatimElevation: 1,033 m; decimalLatitude: -18.7051; decimalLongitude: 26.2039; **Identification:** identifiedBy: Sylvain Delabye; dateIdentified: 2019; **Event:** samplingProtocol: Light-trapping; eventDate: 13/12/2017; **Record Level:** type: PhysicalObject; institutionID: GRBIO URIhttp://grbio.org/cool/8t1f-g2z6; institutionCode: ZMJU; basisOfRecord: PreservedSpecimen
- **Type status:** Other material. **Occurrence:** individualCount: 1; sex: male; lifeStage: adult; **Taxon:** scientificName: Remigiodes remigina (Mabille, 1884); **Location:** continent: Africa; country: Zimbabwe; stateProvince: Matabeleland North; locality: Hwange; verbatimElevation: 1,014 m; decimalLatitude: -18.6954; decimalLongitude: 26.1880; **Identification:** identifiedBy: Sylvain Delabye; dateIdentified: 2019; **Event:** samplingProtocol: Light-trapping; eventDate: 13/12/2017; **Record Level:** type: PhysicalObject; institutionID: GRBIO URIhttp://grbio.org/cool/8t1f-g2z6; institutionCode: ZMJU; basisOfRecord: PreservedSpecimen

#### Description

The detailed diagnosis made by Hacker (2016) enabled identification of this species.

#### Distribution

**Figure 5.**
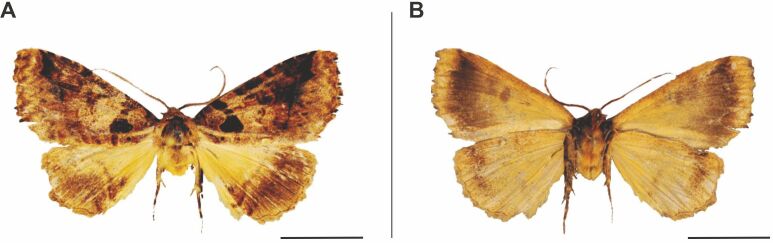
*Remigiodes remigina* (Mabille, 1884), male, Zimbabwe, Hwange. **A.** dorsal view; **B.** ventral view. The scale represents 1 cm.

Our four captured individuals represent the first record of *R. remigina* in Zimbabwe. It is also the first record of *Remigiodes* in the country. This widespread species was already known from the Guinean (Togo, Nigeria), Congolian (Democratic Republic of Congo), Somalian (Ethiopia, Somalia and Kenya) and Zambezian (Tanzania) biogeographic regions and from Madagascar. Our record extended its known continental distribution range by ca. 800 km southwards (Fig. 5).

### Taviodes subjecta

(Walker, 1865)

466EBCD7-1EF9-5AB7-905F-D2238167514C

Erebidae , Pangraptinae

#### Materials

- **Type status:** Other material. **Occurrence:** individualCount: 1; lifeStage: adult; **Taxon:** scientificName: Taviodes subjecta (Walker, 1865); **Location:** continent: Africa; country: Namibia; stateProvince: Kavango East; locality: Bwabwata National Park; verbatimElevation: 1,042 m; decimalLatitude: -18.0688; decimalLongitude: 21.6702; **Identification:** identifiedBy: Sylvain Delabye; dateIdentified: 2019; **Event:** samplingProtocol: Light-trapping; eventDate: 20/11/2016; **Record Level:** type: PhysicalObject; institutionID: GRBIO URIhttp://grbio.org/cool/8t1f-g2z6; institutionCode: ZMJU; basisOfRecord: PreservedSpecimen
- **Type status:** Other material. **Occurrence:** individualCount: 1; lifeStage: adult; **Taxon:** scientificName: Taviodes subjecta (Walker, 1865); **Location:** continent: Africa; country: Namibia; stateProvince: Kavango East; locality: Bwabwata National Park; verbatimElevation: 1,044 m; decimalLatitude: -18.0642; decimalLongitude: 21.6784; **Identification:** identifiedBy: Sylvain Delabye; dateIdentified: 2019; **Event:** samplingProtocol: Light-trapping; eventDate: 20/11/2016; **Record Level:** type: PhysicalObject; institutionID: GRBIO URIhttp://grbio.org/cool/8t1f-g2z6; institutionCode: ZMJU; basisOfRecord: PreservedSpecimen
- **Type status:** Other material. **Occurrence:** individualCount: 1; lifeStage: adult; **Taxon:** scientificName: Taviodes subjecta (Walker, 1865); **Location:** continent: Africa; country: Namibia; stateProvince: Kavango East; locality: Bwabwata National Park; verbatimElevation: 1,044 m; decimalLatitude: -18.0592; decimalLongitude: 21.6872; **Identification:** identifiedBy: Sylvain Delabye; dateIdentified: 2019; **Event:** samplingProtocol: Light-trapping; eventDate: 20/11/2016; **Record Level:** type: PhysicalObject; institutionID: GRBIO URIhttp://grbio.org/cool/8t1f-g2z6; institutionCode: ZMJU; basisOfRecord: PreservedSpecimen

#### Description

The identification of this species was based on Pinhey (1975).

#### Distribution

**Figure 6.**
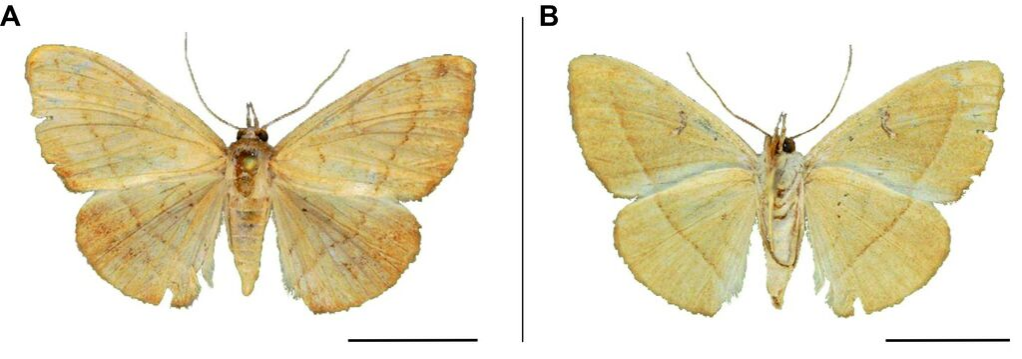
*Taviodes subjecta* (Walker, 1865), male, Namibia, Bwabwata National Park. **A.** dorsal view; **B.** ventral view. The scale represents 1 cm.

We report this species for the first time in Namibia. It was already known from several countries in the Congolian (Democratic Republic of Congo), Somalian (Kenya), Zambezian and Southern African biogeographic regions, including South Africa, Zimbabwe and Zambia, all bordering with Namibia (Pinhey 1975). All three specimens were collected in the Bwabwata National Park, in the Zambezian region (Fig. 6).

### Haplopacha cinerea

Aurivillius, 1905

F031FF90-2248-5205-AB7F-CF2F0D15A291

Lasiocampidae , Lasiocampinae

#### Materials

- **Type status:** Other material. **Occurrence:** individualCount: 1; sex: male; lifeStage: adult; **Taxon:** scientificName: Haplopacha cinerea Aurivillius, 1905; **Location:** continent: Africa; country: Namibia; stateProvince: Kavango East; locality: Bwabwata National Park; verbatimElevation: 1,026 m; decimalLatitude: -18.1118; decimalLongitude: 21.6717; **Identification:** identifiedBy: Sylvain Delabye; dateIdentified: 2019; **Event:** samplingProtocol: Light-trapping; eventDate: 18/11/2016; **Record Level:** type: PhysicalObject; institutionID: GRBIO URIhttp://grbio.org/cool/8t1f-g2z6; institutionCode: ZMJU; basisOfRecord: PreservedSpecimen
- **Type status:** Other material. **Occurrence:** individualCount: 8; sex: 2 females, 6 males; lifeStage: adult; **Taxon:** scientificName: Haplopacha cinerea Aurivillius, 1905; **Location:** continent: Africa; country: Namibia; stateProvince: Kavango East; locality: Bwabwata National Park; verbatimElevation: 1,018 m; decimalLatitude: -18.1170; decimalLongitude: 21.6797; **Identification:** identifiedBy: Sylvain Delabye; dateIdentified: 2019; **Event:** samplingProtocol: Light-trapping; eventDate: 18/11/2016; **Record Level:** type: PhysicalObject; institutionID: GRBIO URIhttp://grbio.org/cool/8t1f-g2z6; institutionCode: ZMJU; basisOfRecord: PreservedSpecimen
- **Type status:** Other material. **Occurrence:** individualCount: 1; sex: male; lifeStage: adult; **Taxon:** scientificName: Haplopacha cinerea Aurivillius, 1905; **Location:** continent: Africa; country: Namibia; stateProvince: Kavango East; locality: Bwabwata National Park; verbatimElevation: 1,009 m; decimalLatitude: -18.1237; decimalLongitude: 21.6862; **Identification:** identifiedBy: Sylvain Delabye; dateIdentified: 2019; **Event:** samplingProtocol: Light-trapping; eventDate: 18/11/2016; **Record Level:** type: PhysicalObject; institutionID: GRBIO URIhttp://grbio.org/cool/8t1f-g2z6; institutionCode: ZMJU; basisOfRecord: PreservedSpecimen
- **Type status:** Other material. **Occurrence:** individualCount: 1; sex: male; lifeStage: adult; **Taxon:** scientificName: Haplopacha cinerea Aurivillius, 1905; **Location:** continent: Africa; country: Namibia; stateProvince: Kavango East; locality: Bwabwata National Park; verbatimElevation: 1,025 m; decimalLatitude: -18.1308; decimalLongitude: 21.6923; **Identification:** identifiedBy: Sylvain Delabye; dateIdentified: 2019; **Event:** samplingProtocol: Light-trapping; eventDate: 18/11/2016; **Record Level:** type: PhysicalObject; institutionID: GRBIO URIhttp://grbio.org/cool/8t1f-g2z6; institutionCode: ZMJU; basisOfRecord: PreservedSpecimen
- **Type status:** Other material. **Occurrence:** individualCount: 1; sex: male; lifeStage: adult; **Taxon:** scientificName: Haplopacha cinerea Aurivillius, 1905; **Location:** continent: Africa; country: Namibia; stateProvince: Kavango East; locality: Bwabwata National Park; verbatimElevation: 1,017 m; decimalLatitude: -18.1375; decimalLongitude: 21.6990; **Identification:** identifiedBy: Sylvain Delabye; dateIdentified: 2019; **Event:** samplingProtocol: Light-trapping; eventDate: 18/11/2016; **Record Level:** type: PhysicalObject; institutionID: GRBIO URIhttp://grbio.org/cool/8t1f-g2z6; institutionCode: ZMJU; basisOfRecord: PreservedSpecimen
- **Type status:** Other material. **Occurrence:** individualCount: 1; sex: female; lifeStage: adult; **Taxon:** scientificName: Haplopacha cinerea Aurivillius, 1905; **Location:** continent: Africa; country: Namibia; stateProvince: Khomas Region; locality: Namibgrens; verbatimElevation: 1,769 m; decimalLatitude: -23.6517; decimalLongitude: 16.2934; **Identification:** identifiedBy: Sylvain Delabye; dateIdentified: 2019; **Event:** samplingProtocol: Light-trapping; eventDate: 4/11/2016; **Record Level:** type: PhysicalObject; institutionID: GRBIO URIhttp://grbio.org/cool/8t1f-g2z6; institutionCode: ZMJU; basisOfRecord: PreservedSpecimen
- **Type status:** Other material. **Occurrence:** individualCount: 3; sex: males; lifeStage: adult; **Taxon:** scientificName: Haplopacha cinerea Aurivillius, 1905; **Location:** continent: Africa; country: Namibia; stateProvince: Otjozondjupa Region; locality: Grootfontein; verbatimElevation: 1,210 m; decimalLatitude: -19.2951; decimalLongitude: 18.7968; **Identification:** identifiedBy: Sylvain Delabye; dateIdentified: 2019; **Event:** samplingProtocol: Light-trapping; eventDate: 15/11/2016; **Record Level:** type: PhysicalObject; institutionID: GRBIO URIhttp://grbio.org/cool/8t1f-g2z6; institutionCode: ZMJU; basisOfRecord: PreservedSpecimen
- **Type status:** Other material. **Occurrence:** individualCount: 1; sex: males; lifeStage: adult; **Taxon:** scientificName: Haplopacha cinerea Aurivillius, 1905; **Location:** continent: Africa; country: Namibia; stateProvince: Otjozondjupa Region; locality: Grootfontein; verbatimElevation: 1,219 m; decimalLatitude: -19.3909; decimalLongitude: 18.8282; **Identification:** identifiedBy: Sylvain Delabye; dateIdentified: 2019; **Event:** samplingProtocol: Light-trapping; eventDate: 16/11/2016; **Record Level:** type: PhysicalObject; institutionID: GRBIO URIhttp://grbio.org/cool/8t1f-g2z6; institutionCode: ZMJU; basisOfRecord: PreservedSpecimen

#### Description

The genitalia dissection and the diagnosis of this species made by Dupont et al. (2016) enabled identification of our specimens.

#### Distribution

**Figure 7.**
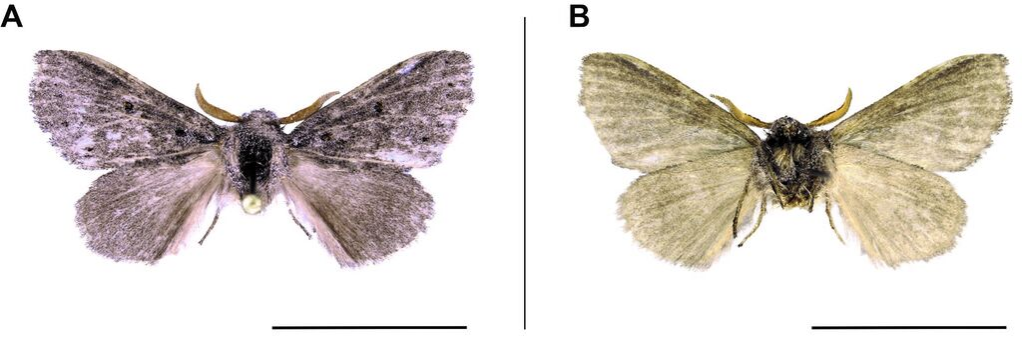
*Haplopacha cinerea* Aurivillius, 1905, male, Namibia, Bwabwata National Park. **A.** dorsal view; **B.** ventral view. The scale represents 1 cm.

Our specimens represented the first record of this species in Namibia. We collected them in the Zambezian (Bwabwata National Park) and Southern African (Namibgrens, Grootfontein) regions. This species was previously reported from these two biogeographical regions only, with records from numerous countries from Tanzania to South Africa. In South Africa, the species was known only from the north-eastern parts of the country (Dupont et al. 2016). Hence, our records substantially extended its known distribution range westwards (Fig. 7).

### Laeliopsis punctuligera

Aurivillius, 1911

34751FE0-25A7-5BBF-9697-5A9ADA474399

Lasiocampidae , Lasiocampinae

#### Materials

- **Type status:** Other material. **Occurrence:** individualCount: 1; sex: male; lifeStage: adult; **Taxon:** scientificName: Laeliopsis punctuligera Aurivillius, 1911; **Location:** continent: Africa; country: Namibia; stateProvince: Kavango East; locality: Bwabwata National Park; verbatimElevation: 1,018 m; decimalLatitude: -18.1170; decimalLongitude: 21.6797; **Identification:** identifiedBy: Sylvain Delabye; dateIdentified: 2019; **Event:** samplingProtocol: Light-trapping; eventDate: 18/11/2016; **Record Level:** type: PhysicalObject; institutionID: GRBIO URIhttp://grbio.org/cool/8t1f-g2z6; institutionCode: ZMJU; basisOfRecord: PreservedSpecimen

#### Description

The identification of this species was based on Pinhey (1975).

#### Distribution

**Figure 8.**
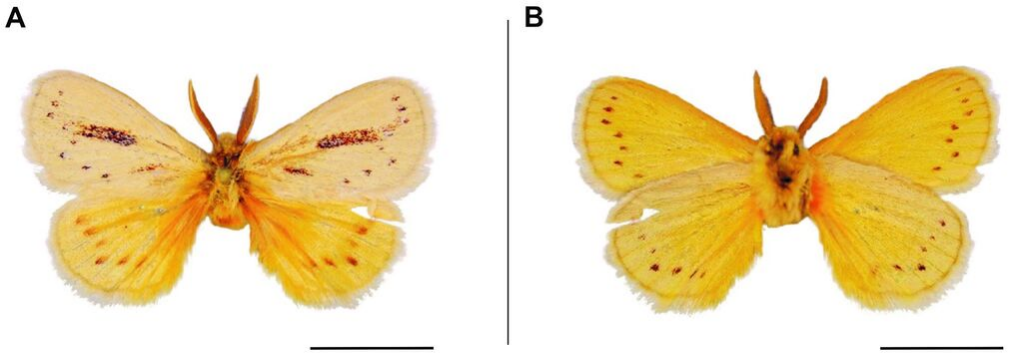
*Laeliopsis punctuligera* Aurivillius, 1911, male, Namibia, Bwabwata National Park. **A.** dorsal view; **B.** ventral view. The scale represents 1 cm.

Our single specimen is the first record of this species in Namibia. It was collected in the Bwabwata National Park, belonging to the Zambezian biogeographical region. The species was previously known from only this region, including from Zambia and Zimbabwe bordering with Namibia. Therefore, extension of its distribution into the Caprivi Strip in north-eastern Namibia is not surprising (Fig. 8).

## Discussion

We presented records of six species of moths (*Plecoptera sarcistis*, *Hypopyra africana*, *Remigiodes remigina*, *Taviodes subjecta*, *Haplopacha cinerea* and *Laeliopsis punctuligera*) newly reported to occur in one or the other of the two sampled countries. Altogether, we reported four species so far not recorded in Namibia and two species so far not recorded in Zimbabwe. Additionally, the genus *Remigiodes* was reported for the first time in Zimbabwe.

All of these species could have been expected in the reported countries because they were previously known to occur in either the Southern African or Zambezian region, whilst four species (except *R. remigina* and *L. punctuligera*) occur in both. All but *R. remigina* were already reported from one or several adjacent countries.

Even though most of the presented new country records could have been expected, at least two of our findings substantially extend the species’ known distribution. The nearest known distribution of *R. remigina* prior to our study was from the Democratic Republic of the Congo. Therefore, our records extend its known distribution by ca. 800 km southwards, although still within the Zambezian geographic region. *Haplopacha cinerea* was previously known from both studied regions. Still, our records from central Namibia extended its known distribution by ca. 600 km westwards.

We consider the presented new country reports as additional evidence of the insufficient knowledge of moth diversity in the Afrotropics. Although the result of a relatively non-intensive sampling effort, our records did increase the number of moth taxa recorded in both countries. Since those records either extend the known distribution of particular species or fill gaps in their continuous distribution, we consider reporting of moth records even from such non-intensive sampling as interesting and highly important for improving our knowledge of diversity and distribution of south African moths.

## Supplementary Material

XML Treatment for Plecoptera sarcistis

XML Treatment for Hypopyra africana

XML Treatment for Remigiodes remigina

XML Treatment for Taviodes subjecta

XML Treatment for Haplopacha cinerea

XML Treatment for Laeliopsis punctuligera

## Figures and Tables

**Figure 2a.**
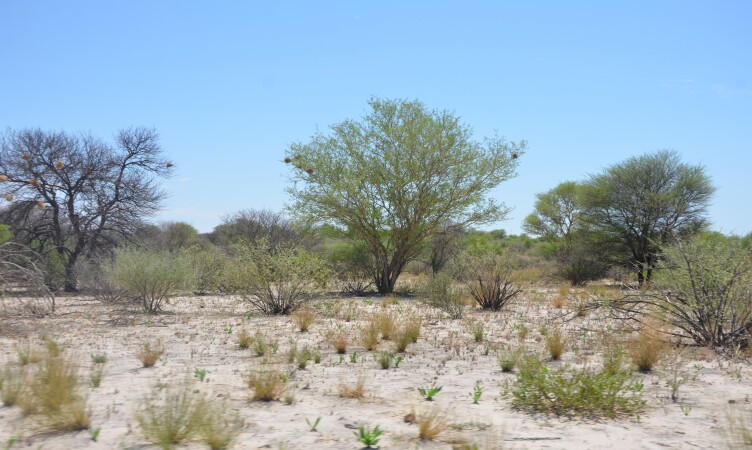
Mosaics of *Vachellia* woodland, mixed with mopane (*Colophospermum mopane*) and *Baikiaea* and microphyllous savannahs (Grootfontein).

**Figure 2b.**
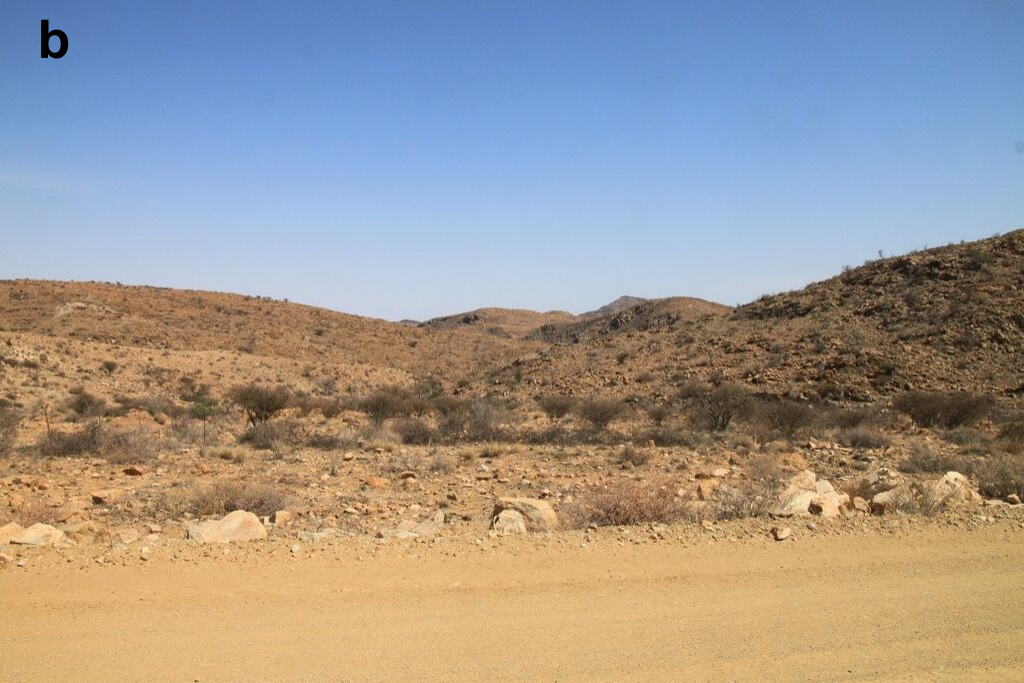
Dry savannahs and shrubs with scattered trees (Namibgrens).

**Figure 2c.**
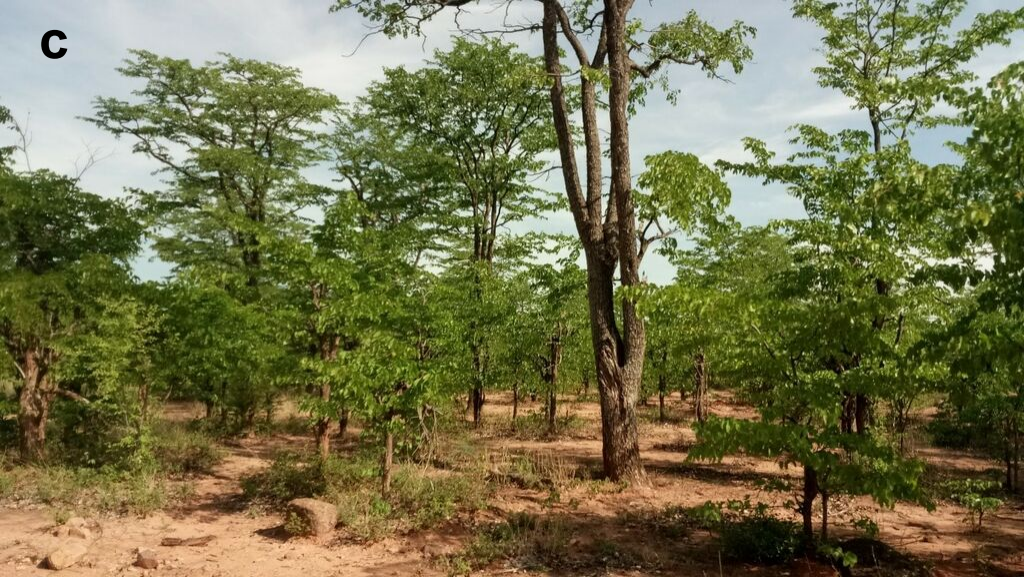
Mosaic of miombo woodland, savannahs and shrubs and mopane (*C. mopane*) woodlands (Hwange).

**Figure 2d.**
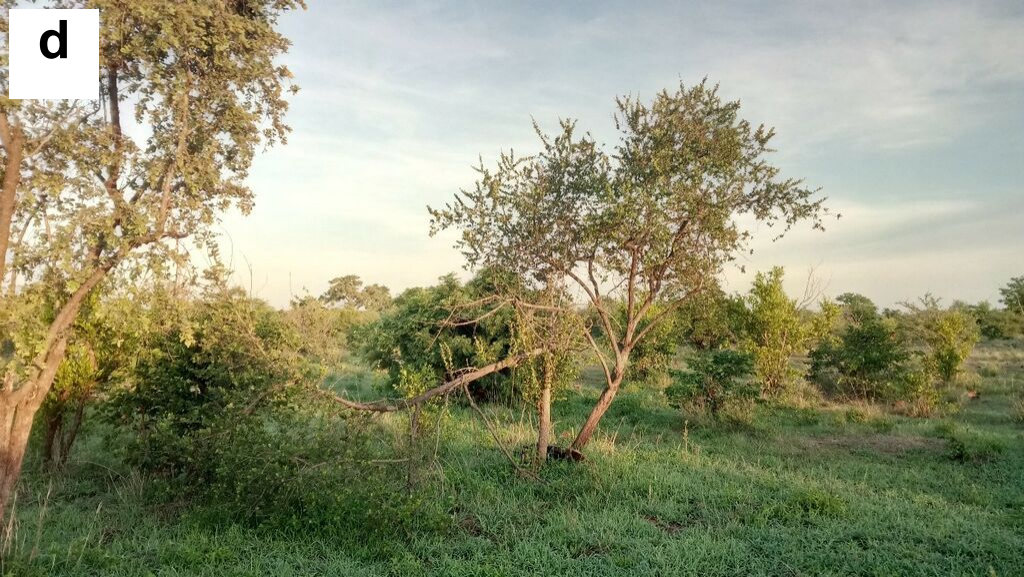
Mosaic of mopane (*C. mopane*) woodland, grasslands and *Baikiaea plurijuga* forests (Bwabwata National Park, Victoria Falls).

**Table 1.** List of the localities and habitats where the moth specimens have been collected.

**Locality**	**Country**	**Biogeographical region**	**Sampling dates**	**Altitude (m a.s.l.)**	**Habitat**
Bwabwata National Park	Namibia	Zambezian	18-20/11/2016	1,050	mosaic of mopane woodland, secondary grasslands and *Baikiaea plurijuga* forests
Grootfontein	Namibia	Southern African	15/16/2016	1,200	mosaics of *Vachellia* woodland, mixed with mopane*Colophospermum mopane* (Fabaceae) and *Baikiaea* (Lamiaceae), and microphyllous savannahs
Namibgrens	Namibia	Southern African	04/11/2016	1,150 to 1,800	dry savannahs and shrubby areas with scattered trees
Hwange	Zimbabwe	Zambezian	13/12/2017	950	mosaic of miombo woodland, savannahs and shrubs and mopane (*Colophospermum mopane*) woodlands
Victoria Falls	Zimbabwe	Zambezian	13-14/12/2017	900	mosaic of mopane woodland, secondary grasslands and *Baikiaea plurijuga* forests

## References

[B6148572] Burgess N., Underwood E., Dinerstein E., Olson D., Itoua I, Schipper J., Ricketts T., Newman K. (2004). Terrestrial ecoregions of Africa and Madagascar: a conservation assessment.

[B6148620] De Prins J., De Prins W. Afromoths, online database of Afrotropical moth species (Lepidoptera).. http://www.afromoths.net/.

[B6371573] Dupont Steen, Simonsen T. J., Zilli Alberto (2016). Haplopacha (Lepidoptera: Lasiocampidae) reviewed: Four new species, first descriptions of the genitalia of both sexes, and unique alar scale organs. Zootaxa.

[B6371600] GBIF.org GBIF Home Page. https://www.gbif.org.

[B6371564] Hacker H. H. (2016). Systematic and illustrated catalogue of the Macroheterocera and Cossoidea Leach,[1815], Zygaenoidea Latreille, 1809, Thyridoidea Herrich-Schäffer, 1846 and Hyblaeoidea Hampson, 1903 of the Arabian Peninsula, with a survey of their distribution (Lepid.). Esperiana.

[B6148628] Hacker H. H. (2019). Moths of Africa. Systematic and illustrated catalogue of the Heterocera (Lepidoptera) of Africa. Volume 1. Biogeography; Boletobiinae (Lepidoptera, Noctuoidea, Erebidae).

[B6371547] Hampson G. F. (1910). Zoological collections from Northern Rhodesia and adjacent territories: Lepidoptera Phalaenae. Proceedings of the Zoological Society of London.

[B6148645] Heppner J. B. (1991). Faunal regions and the diversity of Lepidoptera. Tropical Lepidoptera. Volume 2, Supplement 1.

[B6383632] Kirby W. F. (1896). On a collection of moths from East Africa formed by Dr. W. J. Ansorge, Medical Officer to the Uganda Administration.. Annals and Magazine of Natural History (6).

[B6371539] LepiMAP The atlas of African Lepidoptera. http://lepimap.adu.org.za.

[B6148670] Linder H. Peter, de Klerk Helen M., Born Julia, Burgess Neil D., Fjeldså Jon, Rahbek Carsten (2012). The partitioning of Africa: statistically defined biogeographical regions in sub-Saharan Africa. Journal of Biogeography.

[B6163961] Mittermeier R. A., Robles Gil P., Hoffman M., Pilgrim J., Brooks T., Mittermeier C. G., Lamoreux J., Da Fonseca G. A.B. (2004). Hotspots Revisited.

[B6148684] Murphy Brett P., Andersen Alan N., Parr Catherine L. (2016). The underestimated biodiversity of tropical grassy biomes. Philosophical Transactions of the Royal Society B: Biological Sciences.

[B6148696] Myers Norman, Mittermeier Russell A., Mittermeier Cristina G., da Fonseca Gustavo A. B., Kent Jennifer (2000). Biodiversity hotspots for conservation priorities. Nature.

[B6371514] Pinhey E. G.H. (1975). Moths of Southern Africa.

[B6371505] Ratnasingham S, Hebert P. D.N. (2007). bold: The Barcode of Life Data System (http://www.barcodinglife.org). Molecular Ecology Notes.

[B6148756] Sayre R., Comer P., Hak J., Josse C., Bow J., Warner H., Larwanou M., Kelbessa E., Bekele T., Kehl H., Amena R., Andriamasimanana R., Ba T., Benson L., Boucher T., Brown M., Cress J., Dassering O., Friesen B., Gachathi F., Houcine S., Keita M., Khamala E., Marangu D., Mokua F., Morou B., Mucina L., Mugisha S., Mwavu E., Rutherford M., Sanou P., Syampungani S., Tomor B., Vall A., Vande Weghe J., Wangui E., Waruingi L. (2013). A new map of standardized terrestrial ecosystems of Africa.

